# Characterization of mild or asymptomatic patient admitted with Omicron variant of COVID-19 infection in Tibetan mobile cabin hospital China, August—October 2022

**DOI:** 10.3389/fpubh.2023.1174944

**Published:** 2023-08-09

**Authors:** Fei Shao, Bo Li, Ju-ju Shang, Wen-bin Liu, Hong-bing Wang, Qing-quan Liu

**Affiliations:** ^1^Beijing Hospital of Traditional Chinese Medicine, Capital Medical University, Beijing, China; ^2^Beijing Institute of Traditional Chinese Medicine, Beijing, China; ^3^Beijing Evidence-Based Chinese Medicine Center, Beijing, China; ^4^School of Life Sciences, Beijing University of Chinese Medicine, Beijing, China; ^5^Lhasa People's Hospital, Lhasa, China

**Keywords:** COVID-19, Omicron, Tibetan mobile cabin hospitals, clinical observations, mild or asymptomatic patient

## Abstract

**Background:**

Prior to August 7, 2022, there had been no positive cases of novel coronavirus in Tibet for 920 consecutive days. However, with the first case of Omicron variant infection, the disease rapidly spread and was prevalent in Tibet for nearly 3 months, from August 7th to November 1st. With the spread of the epidemic, the local government responded quickly and established several mobile cabin hospitals to treat patients with mild and asymptomatic Omicron infection. However, the epidemiological and clinical characteristics of these patients are unknown.

**Methods:**

This is a retrospective study including a total of 14,264 mild and asymptomatic cases with Omicron infection in Tibet between August to October, 2022. The clinical data and epidemiological characteristics of COVID-19 cases admitted to Tibet mobile cabin hospitals were collected by using standardized forms from mobile cabin hospital database system, including demographic characteristics, onset symptoms, medication use, past medical history, hospitalization time, and discharge time. In terms of statistical analysis, multivariate Cox regression model was used to analyze the relationship between case characteristics and the length of stay in hospital.

**Results:**

Among 14,264 patients infected with Omicron, the average length of hospital stay was six (4–8, Interquartile range) days. Fifty percent of the patients were discharged by the 6th day, and 90% were discharged by the 10th day. Patients of all ages are generally susceptible to COVID-19, and there was no difference in discharge time, but the average length of hospital stay of Tibetan patients with COVID-19 was longer than that of Han patients. According to the statistics of clinical symptoms, sore throat (38.7%) and fever (19.4%) were the most common symptoms, while muscle pain (17.4%), cough (16.6%), and expectoration (13.2%) were also common. In addition, patients with chronic gastritis had significantly longer hospital stays.

**Conclusion:**

Based on the experience of Tibet mobile cabin hospitals and data analysis, we believe that patients of all ages are generally susceptible to Omicron. Compared with other novel coronavirus strains, Omicron infected patients had a shorter hospital stay, and treatment of symptoms is expected to shorten the time of nucleic acid negative conversion.

## 1. Introduction

Since the discovery of the coronavirus disease 2019 (COVID-19) at the end of 2019, the severe acute respiratory syndrome coronavirus 2 (SARS-CoV-2) has been continuously mutating, with the Omicron variant strain globally dominant at present, whereas the previously prominent alpha, beta, gamma, and delta strains now account for a relatively smaller proportion of the circulating strains. As one of the important variants of COVID-19, Omicron is a strain that specializes in immune evasion, with discrete and rapid transmission processes, and a lower pathogenicity than that of previous strains; with a certain degree of acquired immunity in the population conferred by vaccination, the symptoms of patients infected with Omicron are generally relatively mild (1, 2), only a few patients with severe symptoms needed treatment in hospital. To date, Omicron mutant strains have evolved into various subtypes, with the virulence of some of these strain subtypes continuously increasing (3). From August 7th to November 1st, 2022, multiple clusters of Omicron outbreaks occurred in the Tibet Autonomous Region. Given that Tibet is located on a plateau and lacks substantial medical services and has low vaccination rates, the rapidly disseminating Omicron outbreak posed a huge challenge to the medical and health systems in the region. Under the strong support of the State Council’s Joint Prevention and Control Mechanism Comprehensive Team, various provinces and cities assisted Tibet in conducting COVID-19 nucleic acid testing and provided outbreak treatment and rescue work.

Mobile cabin hospital originated from military warfare to ensure the medical needs of the frontline personnel. In 2020, in response to the sudden COVID-19 epidemic, China adopted mobile cabin hospitals for the first time. These mobile cabin hospitals can be quickly established on a large-scale at low construction costs, providing isolation, medical care monitoring, basic social life and other functions, which contribute to effectively curbing the spread of the virus (4). Since then, as the COVID-19 spread throughout China, shelter hospitals were adopted in a timely manner. Mobile cabin hospitals are generally large temporary hospitals that have been established in large public places and can centrally admit and treat patients with COVID-19, monitoring their condition, provide basic medical and health services, and effectively halting the spread of the virus. The construction of mobile cabin hospitals has laid an important foundation for the effective control of COVID-19 on multiple occasions in various parts of China and has become one of the core methods in China’s fight against the epidemic for the past 3 years. Since the outbreak of the epidemic in Tibet, local government established a series of effective measures to ensure that people frequently carried out COVID-19 nucleic acid testing. Community staff, medical staff in shelter hospitals, and hospitals have established a standardized referral system to treat patients appropriately. Medical staff doing scientific research in the shelter hospital could better understand the characteristics of the prevalence of COVID-19 in the area. Numerous mobile cabin hospitals have been used in large public places, such as convention and exhibition centers and stadiums, with a focus on treating patients with mild, common, or no symptoms of COVID-19, through the integrated use of traditional Chinese and Western medicine providing a tailored fight against COVID-19 outbreak in China.

## 2. Methods

### 2.1. Study design and data collection

Here, we included a total of 14,264 cases of Omicron infections admitted in a Tibetan mobile cabin hospital from August to October 2022. All cases were diagnosed based on the diagnosis and treatment protocol of the National Health Commission of the People’s Republic of China (9th edition). All data were collected by one of the largest mobile cabin hospitals in Tibet, and all cases were discharged from the cabin hospital following a COVID-19-negative nucleic acid test. All included cases were confirmed to be positive through evaluation by hospital emergency or fever clinic personnel, or community COVID-19 nucleic acid test screening. Patients were sent to the mobile cabin hospital for treatment within 2 days after diagnosis and were required to undergo daily COVID-19 testing and leave the cabin hospital following two consecutive days of COVID-19-negative nucleic acid test results. We collected the demographic, epidemiological, clinical data of cases using standardized forms.

### 2.2. Statistical analysis

Descriptive statistical methods were adopted to analyze the continuous variables and categorical variables for different ethnic cases (Tibetan, Han, and other), respectively. The statistical tests for the normal distribution of data included the Kolmogorov–Smirnov and Shapiro–Wilk tests. The differences between the groups were compared by chi-square test. Multivariate Cox regression model was used to analyze the relationship between case characteristics and the length of time in hospital. All statistical tests were two-sided with a significance level of <0.05. Statistical analysis was performed using GraphPad Prism (La Jolla, CA).

### 2.3. Ethical approval

The Ethics Review Committee of the Beijing hospital of Traditional Chinese Medicine, Capital Medical University provided approval for this study (No. 2022BL02-044-02). Additionally, patients’ personal identifying information was anonymized to ensure privacy.

## 3. Results

**Figure 1:**
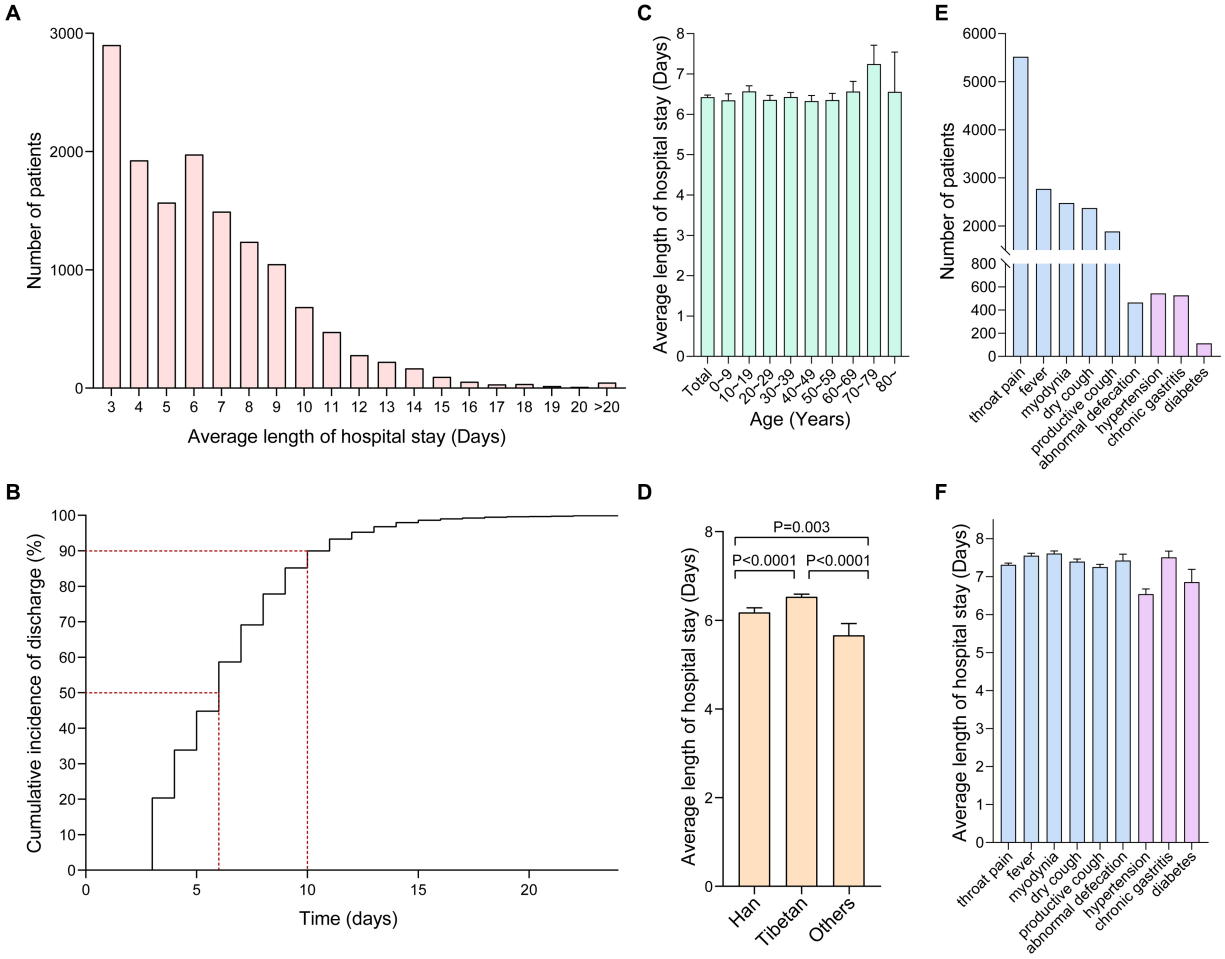
**(A)** The number of patients with COVID-19 with different lengths of hospital stay **(D)** in the Tibetan mobile cabin hospital. **(B)** Percentage of patients with COVID-19 who were discharged from the Tibetan mobile cabin hospital according to the length of hospital stay. Among them, 50% were discharged after 6 days, whereas 90% of them were discharged after 10 days of stay (dashed red lines). **(C)** The length of hospital stay of patients with COVID-19 of different age groups; the panel represents the average length of hospital stay of patients in each age group (the overall average length of hospital stay of patients was 6.43 ± 3.21 days). **(D)** Comparison of the length of hospital stay among Han, Tibetan, and other ethnic groups. **(E)** The number of people who required medication due to different symptoms and underlying medical conditions. **(F)** The average length of hospital stay for patients with different symptoms and underlying medical conditions.

Data showed that the average length of hospital stay for patients infected with the Omicron strain was 6 days (4–8, Interquartile range, IQR; Table 1), which was shorter than the length of hospital stay for patients infected with the Alpha strain at the Wuhan mobile cabin hospital (16.08 ± 5.13 days) in March 2020, or compared with that of patients infected with the Omicron strain at the Shanghai mobile cabin hospital (7.18 ± 3.05 days) in June 2022 (5, 6). Figure 1A shows the number of patients with COVID-19 according to the length of hospital stay, with patients being discharged from the hospital starting from the third day. Figure 1B shows that 50% of patients with COVID-19 were discharged by the sixth day, whereas 90% of them were discharged after 10 days of hospital stay, consistent with the results from the Shanghai mobile cabin hospital. Figure 1C shows the length of hospital stay of patients with COVID-19 according to their age group. In our study, the age range of patients ranged from 1 to 91 years old, and infected patients involved various age groups. In the Shanghai mobile cabin hospital, patients aged 20–29 years old had the fastest recovery. Likewise, in the Tibetan mobile cabin hospital, patients aged 20–29 years old also showed a relatively shorter length of hospital stay (6.36 ± 3.14 days); patients aged 70–79 had longer hospital stays. However, no statistical difference was seen in the discharge time of patients across all age groups. Interestingly, in the Tibet mobile cabin hospital, patients were mainly Tibetans, with Han individuals being only a small portion of patients. Figure 1D shows that the average length of hospital stay of Tibetan patients with COVID-19 was longer than that of Han patients with COVID-19, which might be attributed to the low vaccination rate of Tibetan patients, and/or differences based on ethnicity. Figure 1E shows the number of patients with COVID-19 in the Tibetan mobile cabin hospital with accompanying symptoms and underlying medical conditions, recorded as requiring treatment. Sore throat was one of the main symptoms among patients, with other main symptoms including fever, muscle pain, cough, expectoration, and abnormal bowel movements. Figure 1F shows average length of hospital stay with different symptoms and underlying medical conditions. Only a small proportion (*n* < 600) of patients were recorded to have common underlying medical conditions, such as diabetes, hypertension, and chronic gastritis. To analyze the influence of these factors on the mitigation of COVID-19, we incorporated them into a Cox regression analysis.

**Table 1:** Demographic characteristics and accompanying symptoms of patients with COVID-19.

Characteristics	All cases (*n* = 14,265)	Han (*n* = 10,641)	Tibetan (*n* = 3,228)	Other ethnicity (*n* = 396)	*p-*value
Gender (male)	7464 (52.3%)	5199 (48.9%)	2041 (63.2%)	224 (56.6%)	<0.001
Age (1QR)	32 (21–46)	31 (20–45)	34 (25–47.75)	32 (23–44)	<0.001
Age group (year)					
0–9	1294 (9.1%)	1136 (10.7%)	116 (3.6%)	42 (10.6%)	
10–19	2101 (14.7%)	309 (9.6%)	1756 (16.5%)	36 (9.1%)	
20–29	3118 (21.9%)	803 (24.9%)	2217 (20.8%)	98 (24.7%)	
30–39	3082 (21.6%)	813 (25.2%)	2170 (20.4%)	99 (25.0%)	
40–49	2122 (14.9%)	599 (18.6%)	1461 (13.7%)	62 (15.7%)	
50–59	1551 (10.9%)	513 (15.9%)	990 (9.3%)	48 (12.1%)	
60–69	665 (4.7%)	68 (2.1%)	591 (5.6%)	6 (1.5%)	
70–79	257 (1.8%)	7 (0.2%)	246 (2.3%)	4 (1.0%)	
80–	75 (0.5%)	0 (0.0%)	74 (0.7%)	1 (0.3%)	
Length of hospital stay (days)	6 (4–8)	6 (4–8)	6 (4–8)	5 (3–7)	
Accompanying symptoms					
Fever	2772 (19.4%)	2000 (18.8%)	671 (20.8%)	101 (25.5%)	<0.001
Cough	2372 (16.6%)	1636 (15.4%)	650 (20.1%)	86 (21.7%)	<0.001
Expectoration	1887 (13.2%)	1272 (12.0%)	541 (16.8%)	74 (18.7%)	<0.001
Sore throat	5518 (38.7%)	3954 (37.2%)	1403 (43.5%)	161 (40.7%)	<0.001
Muscle pain	2478 (17.4)	1760 (16.5%)	627 (19.4%)	91 (23.0%)	<0.001
Dyspnea	318 (2.2%)	247 (2.3%)	60 (1.9%)	11 (2.8%)	0.224
Abnormal defecation	465 (3.3%)	313 (2.9%)	136 (4.2%)	16 (4.0%)	0.001
Nausea and vomiting	69 (0.5%)	50 (0.5%)	17 (0.5%)	2 (0.5%)	0.919

Table 2 shows the results of Cox regression analysis. The multivariate model showed that Tibetan people with COVID-19 had a longer length of hospital stay than Han individuals and that the presence of accompanying symptoms that required treatment, such as sore throat, fever, cough, expectoration, and worsening of abnormal bowel movements were unfavorable factors for the relief of COVID-19 among patients.

**Table 2:** Univariate/multivariate Cox analysis of the effect of demographics, symptoms, and underlying medical conditions on the length of hospital stay of patients with COVID-19.

Characteristic	Number of people entering and leaving the cabin (*n* = 14,264)					
	Univariate		Multivariate 1		Multivariate 2	
	Hazard ratio (95% CI)	*P*-value	Hazard ratio (95% CI)	*P*-value	Hazard ratio (95% CI)	*P-*value
Gender (male vs. female) No. 7464 vs. *6801*	1.022 (0.989–1.056)	0.188	1.010 (0.977–1.044)	0.552	0.971 (0.939–1.004)	0.084
Age	0.999 (0.998–1.000)	0.094	0.999 (0.998–1.000)	0.065	1.001 (1.000–1.002)	0.095
Ethnicity						
Tibetan vs. Han No. 10641 vs. *3228*	0.904 (0.869–0.940)	<0.001	0.904 (0.869–0.940)	< 0.001	0.861 (0.828–0.896)	<0.001
Other ethnicity vs. Han No. 396 vs. *3228*	1.161 (1.046–1.289)	0.005	1.160 (1.045–1.288)	0.005	1.176 (1.059–1.306)	0.002
Accompanying symptoms						
Sore throat (with vs. without) No. 5518 vs. *8747*	0.687 (0.664–0.711)	<0.001			0.732 (0.707–0.758)	<0.001
Fever (with vs. without) No. 2772 vs. 11493	0.711 (0.682–0.741)	<0.001			0.828 (0.737–0.931)	<0.001
Muscle pain (with vs. without) No. 2478 vs. *11787*	0.707 (0.677–0.739)	<0.001			0.921 (0.815–1.041)	0.189
Cough (with vs. without) No. 2372 vs. *11893*	0.757 (0.724–0.791)	<0.001			0.846 (0.807–0.886)	<0.001
Expectoration (with vs. without) No. 1887 vs. *12378*	0.796 (0.758–0.835)	<0.001			0.906 (0.861–0.953)	<0.001
Abnormal bowel movements (with vs. without) No. 465 vs. 13800	0.777 (0.708–0.852)	<0.001			0.843 (0.768–0.926)	<0.001
Underlying condition						
Hypertension No. 544 vs. *13721*	0.972 (0.892–1.059)	0.510			0.989 (0.904–1.082)	0.404
Diabetes No. 112 vs. *14153*	0.898 (0.746–1.082)	0.257			0.923 (0.765–1.114)	0.809
Chronic gastritis No. 526 vs. *13739*	0.759 (0.695–0.828)	<0.001			0.824 (0.754–0.900)	< 0.001

## 4. Discussion

During the epidemic of COVID-19 in Tibet, despite the rapid spread of the Omicron variant and the relative shortage of medical resources, medical staff in all parts of China provided rapid support and established several mobile cabin hospitals in a short time to ensure that COVID-19 cases were isolated and treated effectively in a timely manner.

In this study, most cases were mild or asymptomatic. We analyzed COVID-19 cases in the largest mobile cabin hospital in Tibet and found that all age groups were susceptible to Omicron variant infection. Infection with the Omicron strain is associated with a shorter length of hospital stay and relatively faster recovery from symptoms in general, which was also supported by a recent United Kingdom study showing that the risk of hospitalization and mortality from Omicron were approximately 41 and 31% that of Delta, respectively, in the same period (7). At the time of onset, Omicron mainly attacks the respiratory system of the host. While its S protein cannot be effectively cut by transmembrane serine protease 2 in the lungs, rendering its ability to invade the lower respiratory tract lower than that of other strains, Omicron is still able to invade the upper respiratory tract very effectively, leading to fever, sore throat, cough, expectoration, and other symptoms (8). Exacerbation of these symptoms also indicated that viral testing required a longer time to produce a negative result leading to an extended hospital stay. Hence, timely treatment according to symptoms not only helps improve physical discomfort, but also facilitates the yield of a negative result for the Omicron nucleic acid test, thereby reducing the length of hospital stay. Our data show that the proportion of patients who reported underlying medical conditions on their own accord was relatively low, mostly consisting of patients with diabetes, hypertension, and chronic gastritis, potentially leading to data bias. Nevertheless, our analysis suggested that all three underlying medical conditions are potentially deleterious to the length of hospital stay of patients with COVID-19, although a statistical significance was only observed in patients with chronic gastritis. Research has shown that gastrointestinal dysfunction in patients with chronic gastritis or abnormal bowel movements can result in alterations to the gut microbiome and an increase in the levels of inflammatory cytokines, which might affect the prognosis of patients with COVID-19 (9). This suggested that attention should be given not only to the long-term treatment of patients with chronic diseases, but also to those with abnormal bowel movements. Although the variant strain of Omicron is different compared with the original strain used to produce the approved vaccine in China, our results suggest that the vaccine is still effective; thus, the low vaccination rate of Tibetan patients with COVID-19, may have contributed to their longer average hospital stay compared to Han patients.

As of November 1, 2022, the Omicron outbreak in Tibet was coming to an end; however, humanity will still has a long battle with such a cunning virus. Overall, the use of mobile cabin hospitals played a pivotal role in halting the spread of the outbreak and remains one of the key measures employed in various parts of China to overcome multiple rounds of outbreak over the years. As the virulence and pathogenicity of Omicron has gradually weakened, the various outbreak prevention policies of China are continuously optimized, with less large-scale COVID-19 nucleic acid screenings required, and with asymptomatic infections and mild cases being isolated at home, infected people are no longer restricted from purchasing the relevant therapeutic medications, rendering mobile cabin hospitals in China no longer a necessity in the fight against the outbreak. It is plausible that Omicron will continue to infect many people in the future, posing a challenge for China in view of its large population base, aging population, and notable disparity in the allocation of medical resources.

There are also some shortcomings in this study. First, most Omicron-infected individuals in the cabin hospital were mild or asymptomatic, and there is a lack of data support for severe or dying patients. Patients who are referred to the hospital for further treatment due to worsening conditions are also not included in the statistics, because the mobile cabin hospital’s patient diversion strategy and referral system, and the mobile cabin hospital’s statistical data do not include the relevant data of severe patients. Second, the information on Omicron infected individuals in the cabin hospital is not complete, such as vaccination status, nucleic acid CT values, and detailed disease history. But important information about patients was collected and statistically analyzed. It is hoped that this study will provide a basis for the epidemiology of the COVID-19 epidemic and a useful reference for the disease characteristics of mild or asymptomatic Omicron patients.

## 5. Conclusion

Based on the data from Tibetan mobile cabin hospital patients of all ages are susceptible to Omicron infection. Compared with other novel coronavirus strains, Omicron infected patients have a shorter hospital stay. In addition, symptomatic treatment with medications might shorten the time required to yield a COVID-19-negative nucleic acid test result from a previously positive patient. We believe that our report on patients with COVID-19 in Tibetan mobile cabin hospitals will facilitate understanding of the pathogenicity of Omicron and boost knowledge on its infection cycle and accompanying symptoms. As we continue to battle with the COVID-19 pandemic, we recommend that patients infected with the Omicron strain avoid exacerbation of symptoms caused by the infection as much as possible, through the provision of timely symptomatic treatment to eradicate the negative effects of Omicron more rapidly on the human body.

## Data availability statement

The raw data supporting the conclusions of this article will be made available by the authors, without undue reservation.

## Ethics statement

The studies involving human participants were reviewed and approved by the Ethics Review Committee of the Beijing hospital of Traditional Chinese Medicine, Capital Medical University provided approval for this study (No. 2022BL02-044-02). Written informed consent to participate in this study was provided by the participants’ legal guardian/next of kin.

## Author contributions

Q-qL and H-bW conceived and designed the project. FS and BL collected the data. J-jS and W-bL analyzed the data. FS prepared the manuscript. All authors read the manuscript, provided feedback, and approved the final manuscript.

## Funding

This work was supported by the National Multidisciplinary Innovation Team Project of Traditional Chinese Medicine (No. ZYYCXTD-D-202201).

## Conflict of interest

The authors declare that the research was conducted in the absence of any commercial or financial relationships that could be construed as a potential conflict of interest.

## Publisher’s note

All claims expressed in this article are solely those of the authors and do not necessarily represent those of their affiliated organizations, or those of the publisher, the editors and the reviewers. Any product that may be evaluated in this article, or claim that may be made by its manufacturer, is not guaranteed or endorsed by the publisher.
